# Histone Methylation, Energy Metabolism, and Alzheimer's Disease

**DOI:** 10.14336/AD.2024.0899

**Published:** 2025-11-15

**Authors:** Jiaqi Fu, Li An

**Affiliations:** ^1^Department of Nutrition and Food Hygiene, School of Public Health, China Medical University, Shenyang North New Area, Shenyang, Liaoning, China.; ^2^Key Laboratory of Environmental Stress and Chronic Disease Control and Prevention, Ministry of Education, China Medical University, Shenyang, Liaoning, China

**Keywords:** Histone methylation, Energy metabolism, Alzheimer's disease

## Abstract

Alzheimer's disease (AD) is an insidious, progressive, and irreversible neurodegenerative disease characterized by the deposition of extracellular amyloid β-protein (Aβ) to form senile plaques and abnormal phosphorylation of intracellular tau protein to form neuronal fiber tangles. The pathogenesis of AD is complex, and there are several hypotheses, primarily including the Aβ cascade hypothesis, the neurofibrillary tangle hypothesis, the inflammatory hypothesis, and the cholinergic hypothesis. It has been suggested that the dysregulation of multiple energy metabolic pathways, especially mitochondria metabolism, may be related to the severity of AD pathology and disease symptoms in the brain. The modification of histone (lysine) methylation, an actively regulated and reversible process, is closely related to energy metabolism and plays a crucial role in AD development. In summary, histone methylation, energy metabolism, and AD restricted and regulated each other. Here, we review the advances in the correlation between histone methylation, energy metabolism, and AD. This can provide further insights into the mechanisms underlying AD pathogenesis and its control.

## 1. Introduction

Alzheimer's disease (AD) is an insidious, progressive, irreversible, and incurable brain disorder. According to statistics, there were approximately 57 million adults over 40 years of age with AD worldwide in 2019, and this number is expected to grow to 153 million by 2050 [1]. The risk factors of AD mainly include age, genetics, and environment [2, 3]. The pathological features of AD mainly include abnormal deposition of amyloid β-protein (Aβ) and neurofibrillary tangles (NFTs) caused by hyperphosphorylated tau protein. The pathogenesis of AD is complex, primarily including the Aβ cascade hypothesis, the neurofibrillary tangles hypothesis, the inflammation hypothesis, and the cholinergic neuron hypothesis. Patients with AD can be classified into familial and sporadic types. AD is one of the aging-related neurodegenerative diseases. According to the age at diagnosis, AD is classified as early-onset and late-onset AD. Early-onset AD is closely associated with the mutation of Aβ-related genes, such as amyloid precursor protein (APP) and presenilin-1 (PS1). Apolipoprotein E4 (APOE4) is the major genetic risk factor for late-onset AD. With aging, energy metabolism in the body is impaired and the activity of histone methylation-modifying enzymes is altered. López-Otin et al. [4] identified nine hallmarks of aging process, including genomic instability, telomere attrition, epigenetic alterations, loss of proteostasis, mitochondrial dysfunction, cellular senescence, deregulated nutrient sensing, stem cell exhaustion, and altered intercellular communication. Numerous findings have revealed that these hallmarks are all closely related to AD. Mitochondria is the center of energy metabolism in the body, and its dysfunction leads to severely impaired energy metabolism. Epigenetics modifications include DNA methylation, histone modifications, chromatin remodeling, and RNA modifications. Histone modifications play important roles in epigenetic modifications, among which histone methylation is a more complex, enduring, and stable modification. There is growing evidence that the dysregulation of multiple energy metabolic pathways may be associated with the pathological changes in AD brain and the severity of disease symptoms. In addition, histone methylation is a prevalent epigenetic modification closely related to energy metabolism and has emerged as an essential player in the onset and development of AD. Besides, a large number of studies have shown that energy metabolism and histone methylation are mutually regulated, but neither has been fully elucidated in relation to AD. Therefore, understanding the interconnection between histone methylation, energy metabolism, and AD will provide further insight into the mechanisms of AD pathogenesis and its control.

## 2. Histone methylation and AD

### 2.1 Histone methylation

The nucleosome is the basic structural unit of chromatin and consists of histone octamer and double-stranded DNA. Histone octamer is composed of four different histone proteins: H2A, H2B, H3, and H4. Various covalent modifications, including methylation, phosphorylation, acetylation, and ubiquitination, occur at the ends of histones. These modifications can regulate gene expression by directly changing the interaction between histones and DNA, or by recruiting proteins or protein complexes with specific functions. These post-translational modifications of histones are also called the “histone code” because the histone modification sites and their interactions with other regulatory proteins form a network that regulates gene expression. It is worth noting that histone modification sites do not function independently but interact with and regulate each other. Histone post-translational modifications play an essential role in epigenetic regulation and are closely related to the regulation of gene expression. Different modifications exhibit different characteristics. For example, histone methylation modifications are relatively stable, whereas acetylation modifications are highly dynamic, and phosphorylation, ubiquitination, and other modifications are unstable. Histone methylation can occur in the lysine (Lys or K) and arginine residues. Owing to the diversity of modification sites and the fact that the same modification site can be methylated to different degrees, methylation is a more complex modification than other post-translational modifications of histones. Lysine can be mono-methylated (me1), di-methylated (me2), or tri-methylated (me3), while arginine can be mono-methylated and symmetrically or asymmetrically di-methylated. Depending on the methylation site, lysine methylation of H3 and H4 can regulate transcriptional activation and repression. Methylation of histone 3 lysine 4 (H3K4), H3K36, and H3K79 is primarily involved in gene activation, and methylation of H3K9, H3K27, and H4K20 is mainly associated with transcriptional repression. Arginine methylation has been found to promote transcriptional activation.

**Figure 1.**
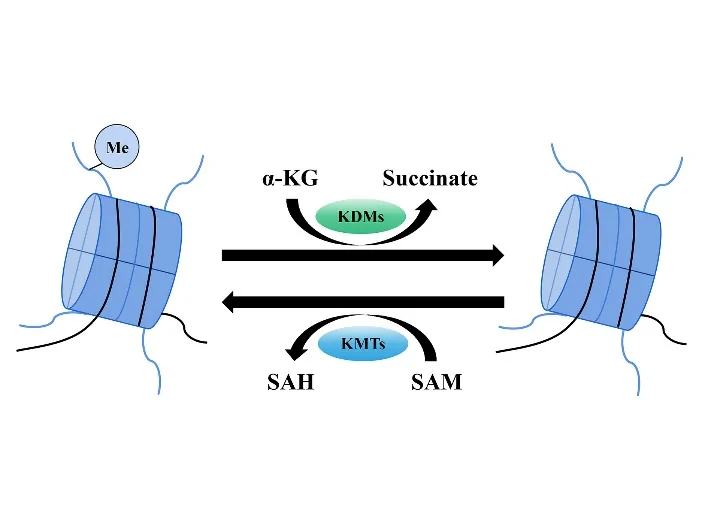
**Histone methylation and demethylation process**. Note: Histone methylation refers to the transfer of methyl groups to the lysine residues of histones catalyzed by KMTs, with SAM as the methyl donor, whereas SAM itself becomes SAH. Histone demethylation refers to the change of histone modification with α-KG as the substrate and the removal of methyl groups from the lysine residues catalyzed by KDMs, while α-KG becomes succinate. SAM: s-adenosyl-L-methionine; SAH: s-adenosyl-L-homocysteine; α-KG: α-ketoglutarate; Me: methyl.

Histone methylation is an actively regulated and reversible process, among which histone (lysine) methylation modification has been most commonly studied. It involves histone lysine methyltransferases (KMTs)-mediated methylation and histone lysine demethylases (KDMs)-mediated demethylation (Fig. 1). Based on their catalytic domain sequence, KMTs are classified into SET-containing and non-SET-containing domains, of which only KMT4 (DOT1L) (Table 1) is a non-SET-containing methyltransferase. KDMs include lysine-specific demethylases (LSD) and the Jumonji C domain-containing (JMJD) protein family (Table 2). The process of KMTs-mediated histone methylation is intricate, as KMTs become activated only after forming a complex with the corresponding protein and subsequently recognize specific histone modification sites for methylation, thereby regulating gene expression. For instance, pre-existing H3K9me2 or H3K9me3 is specifically recognized by the chromodomain of heterochromatin protein 1, which subsequently recruits the catalytic KMT1A (SUV39H1) to tri-methylate neighboring H3K9 sites, thereby establishing a positive feedback loop [5]. H3K27 methylation represses gene expression by recruiting poly-comb repressive complexes in which the poly-comb is a structural domain that recognizes the H3K27 site [6]. Unlike KDMs, KMTs do not require the involvement of other proteins and function directly.

**Table 1:** Classification and modification Loci of KMTs.

Nomenclature	Official Symbol/Alias	Loci	References
**KMT1A**	SUV39H1	H3K9	[194, 195]
**KMT1B**	SUV39H2	H3K9	[196]
**KMT1C**	G9a/EHMT2	H3K9	[197]
**KMT1D**	GLP/EHMT1	H3K9	[7]
**KMT1E**	SETDB1/ESET	H3K9	[198]
**KMT1F**	SETDB2	H3K4	[199]
**KMT2A**	MLL1	H3K4	[200]
**KMT2B**	MLL2	H3K4	[201, 202]
**KMT2C**	MLL3	H3K4	[203]
**KMT2D**	MLL4	H3K4	[204]
**KMT2E**	MLL5	H3K4	[205]
**KMT2F**	SET1A	H3K4	[206]
**KMT2G**	SET1B	H3K4	[207]
**KMT2H**	ASH1L	H3K4, H3K9, H3K36, H4K20	[208, 209]
**KMT3A**	SETD2	H3K36	[210]
**KMT3B**	NSD1	H3K36, H4K20	[211]
**KMT3C**	SMYD2	H3K4, H3K36	[212, 213]
**KMT3D**	SMYD3	H3K4	[214]
**KMT3E**	WHSC1L1/NSD3	H3K4, H3K27, H3K36	[215, 216]
**KMT3F**	WHSC1/NSD2	H3K4, H3K27, H3K36, H4K20	[215, 217, 218]
**KMT4**	DOT1L	H3K79	[219]
**KMT5A**	SET8/PR-SET7/SETD8	H4K20	[220, 221]
**KMT5B**	SUV20H1	H4K20	[222]
**KMT5C**	SUV20H2	H4K20	[222]
**KMT6**	EZH2	H3K27	[223]
**KMT7**	SET7/SET9/SETD7	H3K4	[214, 221]
**KMT8A**	PRDM2/RIZ1	H3K9	[224]
**KMT8B**	PRDM9	H3K4	[225]
**KMT8C**	PRDM6	H4K20	[226]
**KMT8D**	PRDM8	H3K9	[227]
**KMT8E**	PRDM3	H3K9	[228]
**KMT8F**	PRDM16	H3K9	[228]

### 2.2 KMTs and AD

Numerous studies have shown that KMTs are involved in AD pathogenesis by affecting the expression of related proteins and genes.

### 2.2.1 G9a, GLP, and EZH1/2

Synaptic plasticity is recognized as the neurobiological basis of learning and memory and is involved in the development of AD. Brain-derived neurotrophic factor (BDNF), predominantly synthesized in the brain and abundant in the cerebral cortex and hippocampus, regulates long-term potentiation (LTP) which is one of the most widely cited cellular models underlying synaptic plasticity. Glutamate receptors are also involved in synaptic plasticity. They are classified as ionotropic and metabotropic types, which include the N-methyl-D-aspartic receptor (NMDAR) and the α-amino-3-hydroxy-5-methyl-4-isoxazole propionic acid receptor. G9a and GLP are histone methyltransferases that mono- and di-methylate H3K9. They share approximately 80% sequence identity in the SET-containing domain to form a heterodimer [7]. Reportedly, BDNF levels are reduced in patients with AD [8, 9]. Sharma et al. [10] demonstrated that the inhibitors of G9a and GLP, such as BIX01294 and UNC0642, upregulated BDNF expression in the CA1 region of acute hippocampal slices from five- to seven-week-old male Wistar rats treated with Aβ_1-42_ oligomers and further maintained late-LTP. In late-stage five familial AD mutations mice, it has been reported that the inhibitors of G9a and GLP rescued cognitive deficits by relieving H3K9me2-mediated repression of glutamate receptors transcription [11]. In addition, the knockdown of G9a was found to reverse the reduction of hypoxia-induced neprilysin (NEP) in mouse primary cortical and hippocampal neurons, facilitating Aβ clearance [12]. In the prefrontal cortex of the P301S Tau mouse model, Wang et al. [13] observed that UNC0642 had an effect on improving spatial and recognition memory in five- to seven-month-old mice (late stage of cognitive impairment associated with NFT deposits) by inhibiting tau protein hyperphosphorylation. However, the mechanism of the effect of the inhibitor on tau protein phosphorylation is still unclear. The cytoskeleton plays vital roles in coordinating neuronal transport and maintaining synaptic function [14]. This study also found that UNC0642 restored approximately 22% of the down-regulated genes (enriched in the regulatory cytoskeleton) in mice, which facilitated the restoration of neuronal excitability and synaptic transmission in the prefrontal cortex and improved cognition [13]. Furthermore, the decrease in autophagy is associated with an increase in the proportion of damaged mitochondria, and consequently leads to increased accumulation of reactive oxygen species (ROS) and Aβ. Autophagosome formation requires the participation of microtubule-associated protein 1 light chain 3 beta, diabetes- and obesity-regulated protein, and WD repeat domain phosphoinositide-interacting protein 1 [15-17]. Studies have shown that G9a inhibits the expression of these proteins, thereby controlling autophagy [18], which may be detrimental to the expression of various aging phenotypes.

Postsynaptic membrane density (PSD) is the structural basis of postsynaptic signal transduction and integration. PSD95, the major scaffolding protein in the PSD, integrates NMDAR signals and regulates synaptic plasticity and learning memory. Griñán-Ferré et al. [19] found that UNC0642 treatment increased PSD95 protein levels. EZH2, a negative regulator of dendritic morphogenesis in mammalian neurons, has been documented to interact with the transcriptional repressor chromodomain Y-like transcription co-repressor to upregulate H3K27me3 levels in the BDNF promoter region, thereby limiting dendritic development [20]. EZH1 and EZH2 play antagonistic roles in the regulation of PSD95 transcription [21], but none have been observed in AD.

**Table 2:** Classification and modification Loci of KDMs.

Nomenclature	Official Symbol/Alias	Loci	References
**KDM1A**	LSD1	H3K4, H3K9	[229, 230]
**KDM1B**	LSD2/AOF1	H3K4	[231]
**KDM2A**	FBXL11/JHDM1A	H3K36	[232]
**KDM2B**	FBXL10/JHDM1B	H3K36	[233]
**KDM3A**	JMJD1A/JHDM2A	H3K9	[234]
**KDM3B**	JMJD1B/JHDM2B	H3K9	[235]
**KDM3C**	JMJD1C/JHDM2C	H3K9	[236]
**KDM4A**	JMJD2A/JHDM3A	H3K9, H3K36	[237]
**KDM4B**	JMJD2B/JHDM3B	H3K9, H3K36	[238]
**KDM4C**	JMJD2C/JHDM3C/GASC1	H3K9, H3K36	[239, 240]
**KDM4D**	JMJD2D/JHDM3D	H3K9, H3K36	[240]
**KDM5A**	JARID1A/RBP2	H3K4	[241]
**KDM5B**	JARID1B/PLU1	H3K4	[242]
**KDM5C**	JARID1C/SMCX	H3K4	[243]
**KDM5D**	JARID1D/SMCY	H3K4	[243]
**KDM6A**	UTX	H3K27	[244, 245]
**KDM6B**	JMJD3	H3K27	[244, 245]
**KDM7A**	JHDM1D/KIAA1718	H3K9, H3K27	[246]
**KDM7B**	JHDM1F/PHF8	H3K9, H3K27, H4K20	[247]
**KDM7C**	PHF2	H3K9, H3K4	[248]
**KDM8**	JMJD5	H3K36	[249]

### 2.2.2 KMT2 family, SMYD3, and SETD7

KMT2A modulates memory by influencing the expression of genes involved in synaptic plasticity [22]. Cao et al. [23] found that administration of the SET1/MLL inhibitor WRD5-0103 inhibited the increase in H3K4me3 levels and the down-regulation of glutamatergic synaptic receptor expression, and restored synaptic plasticity in the prefrontal cortex, thus alleviating cognitive deficits in P301S Tau mice and five familial AD mutations mice. Among the up-regulated genes reversed by WRD5-0103 treatment in the prefrontal cortex of AD mice, many showed increased H3K4me3 enrichment at their promoters, including serum- and glucocorticoid-regulated kinase 1 (SGK1) and early growth response 1 (EGR1). Evidence showed that SGK1, a member of the serine/threonine protein kinase family, was significantly elevated in the prefrontal cortex of patients with AD. The administration of specific SGK1 inhibitor GSK650394 in AD mice reduced tau protein hyperphosphorylation [23]. Overexpression of SGK1 in the hippocampus was reported to increase Aβ degradation mediated by insulin-degrading enzyme (IDE) and non-amyloid formation of APP mediated by a disintegrin and metalloprotease 10 (ADAM10) (a type of α-secretase), thereby affecting the reduction of Aβ production and deposition. However, the amyloid formation of APP, mediated by β-secretase 1 (BACE1), was not affected [24]. EGR1 is a member of the immediate early gene family. It has been reported that EGR1 is associated with Aβ production, tau protein phosphorylation [25, 26], and synaptic plasticity [27, 28], but whether EGR1 is a deleterious or protective factor in the AD is still controversial and needs further explored. SETDB1 (ESET) was found to upregulate the level of H3K9me3 in the striatum, which consequently decreased the expression of EGR1, ultimately causing impaired neurological function in the striatum in an R6/2 mouse model of Huntington's disease, resulting in various behavioral deficits [29]. A recent study has discovered that SETDB1 (ESET) inhibitors decreased H3K9me3 levels, and improved motor function and neuropathological symptoms in Huntington's chorea transgenic mice [30]. Given that EGR1 has been implicated in the pathological changes in AD and can be modulated by SET1/MLL and SETDB1 (ESET) through the methylation pathway, we hypothesize that the two histone methyltransferases may also influence the pathological progression of AD by affecting EGR1.

In addition, a recent study has demonstrated that the histone methyltransferase SMYD3, which primarily catalyzes H3K4me2/3, was significantly elevated in the prefrontal cortex of AD patients and P301S Tau mice. BCI-121, the inhibitor of SMYD3, has been found to rescue NMDAR and cognitive deficits in P301S Tau mice [31]. In a study conducted by Bichmann et al. [32] showed that knock down and inhibitor studies supported by proteomics data led to the identification of SETD7 as a novel lysine methyltransferase for tau. The above study also revealed that SETD7 specifically methylated tau at K132, an event that facilitated subsequent methylation at K130.

### 2.2.3 Others

In the hippocampus of aged mice, ETP69 (an inhibitor of SUV39H1) was demonstrated to decrease the H3K9me3 level of the BDNF promoter [33]. In addition, PRDM5, which is associated with astrocyte proliferation and neuronal apoptosis, may be involved in inflammatory processes in the central nervous system [34]; however, these have not been observed in AD.

Apart from the KMTs inhibitors mentioned above, a large number of specific inhibitors of different KMTs have been developed over the past few years. Among the KMTs inhibitors, the inhibitors of EZH2 and DOT1L have entered the stage of clinical trials. For example, tazemetostat (EPZ6438) is an oral EZH2 inhibitor that can be used to treat sarcoma [35]. Valemetostat is the first dual EZH1/2 inhibitor approved for the treatment of adult T-cell leukemia/lymphoma [36]. Besides, pinometostat (EPZ5676), the inhibitor of DOT1L, is being studied and tested for the treatment of diseases such as MLL and AML leukemia [37]. Current studies mainly focus on the role of these inhibitors in the treatment of some cancers and many blood malignancies, but whether they have effects on the development of AD has not yet been reported.

### 2.3 KDMs and AD

There are two main types of KDMs, including flavin adenine dinucleotide (FAD)-dependent lysine-specific demethylases (KDM1A/1B or LSD1/2) and JmjC domain-containing lysine demethylases. The latter has diverse catalytic substrates that require both α-KG and divalent iron ions (Fe^2+^) and has the potential to remove trimethyl groups from the histone lysine residues.

### 2.3.1 LSD

LSD, the first histone demethylase discovered, belongs to the superfamily of FAD-dependent amine oxidases. It specifically removes mono- and di-methylated modifications of H3K4 *in vitro* and mono- and di-methylated modifications of H3K9 in vivo with the participation of FAD, but fails to demethylate H3K4me3. It has been demonstrated that increased LSD1-mediated histone demethylation attenuated tau protein-mediated neurodegeneration in PS19 Tau mice that mimic tau protein aggregation [38]. Longaretti et al. [39] indicated that LSD1/neuroLSD1 (a neuron-specific splice variant) levels in the hippocampus decreased significantly with aging in individuals aged 20-94 years, regardless of sex. Additionally, LSD1 was found to influence synaptic plasticity-related proteins in neuroLSD1^KO^ mice under environmental stress conditions. However, the impact of LSD1 on AD remains unclear and warrants further exploration.

The KDMs inhibitors, which are similar to KMTs inhibitors, are still in the research phases. Pharmacological inhibition of LSD1 has shown promising therapeutic benefits of AD. ORY-2001, one of the LSD1 inhibitors, has completed the phase Ⅱa clinical trial in patients diagnosed with mild to moderate AD and undergone the phase Ⅱa clinical trial in patients diagnosed with moderate to severe AD [40, 41]. Besides, ORY-2001 has been reported to rescue cognitive function and reduce the inflammation signature in the SAMP8 (senescence accelerated mouse prone 8) mice, which is used to study AD [42].

### 2.3.2 The Jumonji C domain-containing (JMJD) family histone demethylases

The JMJD family of demethylases consists primarily of the KDM1~KDM7 family. The KDM2 family, including KDM2A/JHDM1A and KDM2B/JHDM1B, participates in the demethylation of H3K4me3 and H3K36me1/2. It is highly expressed in the forebrain, cerebellum, occipital lobe, and postcentral gyrus during early mammalian embryonic development, and changes with developmental stage and age [43]. Moreover, mutations and abnormal expression of these genes can alter the transcriptional levels of target genes by affecting their demethylation, thereby regulating the expression of downstream signaling pathways and affecting neurogenesis, apoptosis, oxidative stress, and energy metabolism [44-47]. KDM2B affects the formation of neuroinflammatory amyloid plaques in the prodromal phase of AD and is directly involved in AD pathogenesis [48]. The susceptibility genes rs28604990 and rs7955747, which are located within a haplotype block that includes intron 6 and intron 12 of KDM2B, respectively, are most substantially associated with AD in APOE4 non-carriers [49].

**Table 3:** KMTs/KDMs and AD.

KMTs/KDMs	Category	Affected pathways/genes/proteins	Pathologies/phenotypes in AD	Experimental subjects	References
**KMTs**	G9a/GLP (KMT1C/1D)	BIX01294, UNC0642 (G9a, GLP inhibitors) →BDNF, Glutamate receptors (NMDAR), PSD95↑; Hypoxia→H3K9me2 level in the NEP promoter↑→NEP↓ (reversed by G9a knockdown)	Synaptic plasticity↑; Aβ clearance↑; Tau protein hyperphosphorylation↓;	Rats, mice; Cells	[10-13, 19]
**KMTs**	MLL1 (KMT2A)	Genes related to AD and LTP	Synaptic plasticity	Mice	[22]
**KMTs**	SET1/MLL family (SET1A/1B (SETD1A/1B))	WRD5-0103 (SET1/MLL inhibitor) →Synaptic plasticity in prefrontal cortex↑; WRD5-0103→EGR1, SGK1↓; GSK650394 (SGK1 inhibitor) →Hyperphosphorylation of tau protein↓; SGK1↑→IDE↑/ADAM10↑→Aβ degradation↑/Aβ production↓ (BACE1-mediated APP amyloid formation process is not affected)	Synaptic plasticity↑; Aβ degradation↑, Aβ production↓; Tau protein hyperphosphorylation↓	Mice, cells	[23-28]
**KMTs**	SMYD3	BCI-121 (SMYD3 inhibitor) →NMDAR↑	Cognitive function↑	Human tissues, mice, and cells	[31]
**KDMs**	LSD1 (KDM1A)	None	Tau protein phosphorylation↓; Cognitive function↓; Inflammation↑	Mice	[38, 42]
**KDMs**	JHDM1B (KDM2B)	None	Aβ formation↑	Human brain tissues	[49]
**KDMs**	KDM4A (homologous to human JHDM3A)	None	Tau^R406W^-induced locomotion defects	Drosophila	[50]
**KDMs**	JARID1A (KDM5A)	①BACE1↑, sAPPβ↑; ②p-tau↑; ①SOD1↑, 4-HNE↑, Aldh2↓; ④TNF-α↑, IL-6↑; ⑤SYP↓	①Aβ↑; ②Tau protein phosphorylation↑; ①Oxidative stress↑; ④Neuroinflammation↑; ⑤Synaptic plasticity↓	Mice	[51]
**KDMs**	JMJD3(KDM6B)	CUR→JMJD3-H3K27me3-BDNF→Aβ aggregation, mitochondrial stress response↓	Aβ aggregation, mitochondrial stress response↓	Mice, cells	[53]

Note: IL-6: interleukin 6; TNF-α: tumor necrosis factor alpha; sAPPβ: soluble amyloid precursor protein β; SOD: superoxidedismutase; 4-HNE: 4-Hydroxynonenal; Aldh2: aldehyde dehydrogenase-2; SYP: synaptophysin; CUR: curcumin (“↑” denotes increased levels; “↓” denotes reduced levels).

According to bioinformatic analysis, the expression levels of JHDM1A, JHDM2A/2B, and JHDM3A/3B were significantly higher in the postmortem brain tissue of AD patients than in non-demented controls, whereas JHDM1B level was downregulated. However, the results need to be further verified by more experiments. In tau^R406W^-induced transgenic Drosophila model, the knockdown of KDM4A (homologous to human JHDM3A) was found to ameliorate tau^R406W^-induced locomotion defects by restoring heterochromatin [50]. Griñán-Ferré et al. [51] discovered that JARID1A (KDM5A) was associated with Aβ in five familial AD mutations mice model. The KDM6 family of H3K27me2/3 demethylases includes JMJD3 and UTX. High expression of JMJD3 has been documented to enhance H3K27me3 demethylation of disstal-less homeobox2, mixed lineage leukemia 1, mammalian achaete-scute homologue-1, and other genes related to neuronal development [52]; however, whether it has this effect on AD is unknown. Evidence showed that medium and high doses of curcumin inhibited Aβ aggregation and attenuated mitochondrial stress response by modulating the JMJD3-H3K27me3-BDNF pathway in APP/PS1 mice and SH-SY5Y cells [53]. In addition, a study in *caenorhabditis elegans* discovered that elevated UTX activated the insulin/IGF-1 signaling pathway (IIS) and prevented the transcription factor DAF-16 from entering the nucleus to activate anti-aging genes. Similar aging alterations have also been observed in brain samples from rhesus macaques [54]. However, whether UTX plays the same role in AD development remains to be confirmed.

Chen et al. [55] found that the mTOR signaling pathway was overactive in the hippocampus of PHF8 knockdown mice, resulting in LTP attenuation and cognitive deficits. However, whether PHF8 affects AD in the way mentioned above remains to be explored.

**Figure 2.**
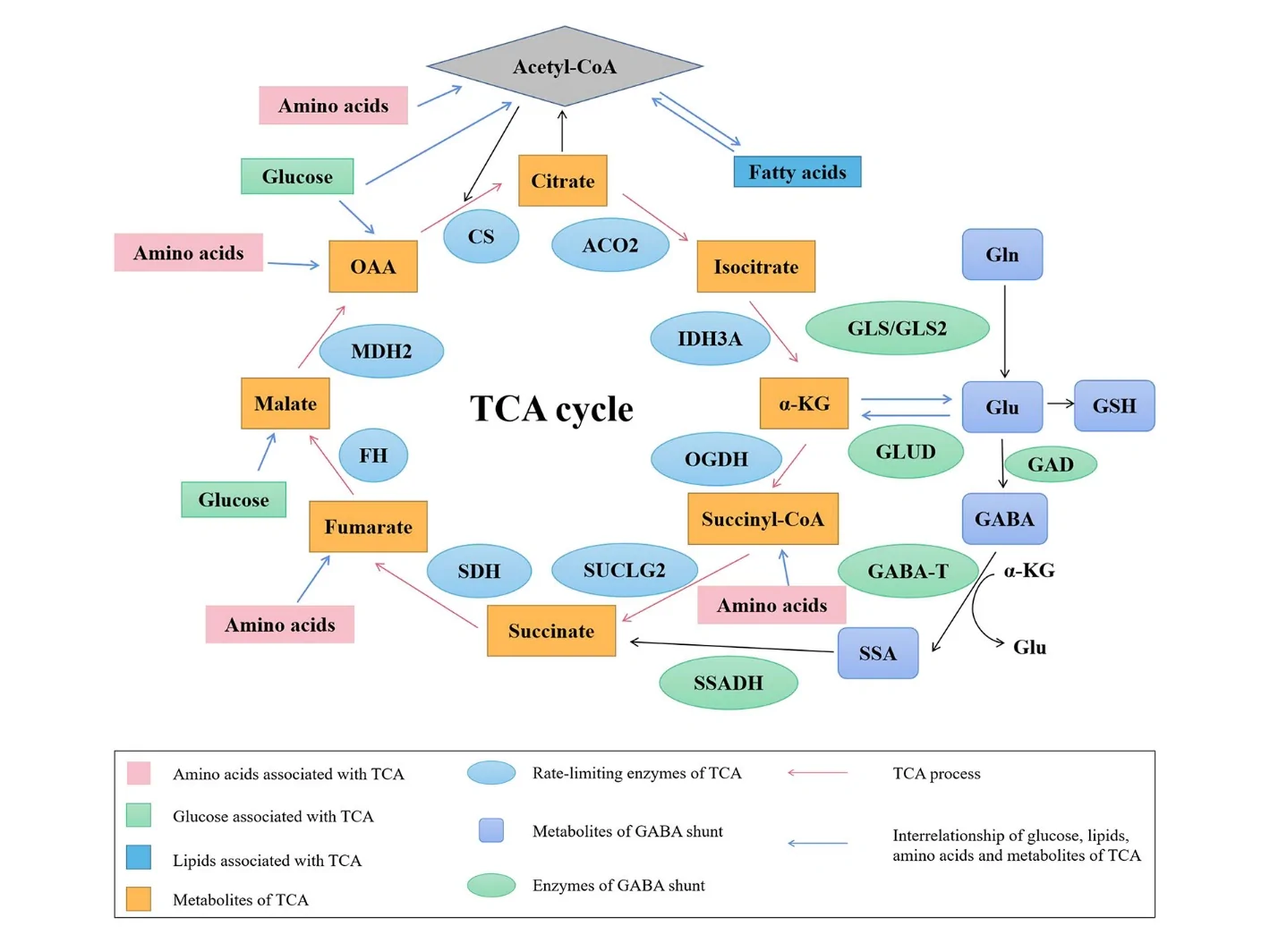
**Glucose, fatty acid, and amino acid metabolism and TCA cycle**. Note: ACO2: aconitase 2; IDH3A: isocitrate dehydrogenase 3A; α-KG: α-ketoglutaric acid; OGDH: α-ketoglutarate dehydrogenase; SUCLG2: succinyl- CoA synthetase; SDH: succinate dehydrogenase; FH: fumarate hydratase; MDH2: malate dehydrogenase 2; OAA: oxaloacetic acid; CS: citrate synthase; Glu: glutamate; Gln: glutamine; GLUD: glutamate dehydrogenase; GLS/GLS2: glutaminase; GSH: glutathione; GAD: glutamic acid decarboxylase; GABA-T: γ-aminobutyric acid transaminase; GABA: γ-aminobutyric acid; SSA: succinic semialdehyde; SSADH: succinate semialdehyde dehydrogenase

Besides, researchers observed changes in other histone methylation marks in AD patients compared with controls, with mainly increased H4K20me2 and H3K79me1 levels and decreased H3K79me2, H3K36me2, H4K20me3, H3K27me1, and H3K56me1 marks [56].

Taken together, KMTs and KDMs have effects on proteins and genes involved in AD-related pathologies, such as synaptic plasticity, Aβ formation and clearance, and tau protein hyperphosphorylation by modifying lysines at different sites on histones (Table 3). For the drugs addressed before, such as EZH2, DOT1L, and LSD1 inhibitors, they are safe and tolerable and most of them have their best clinical response after weeks to months of continuous therapy. However, some problems such as selectivity as well as resistance of single-target drugs and potential off-target effects limit their clinical application. Besides, the inhibitors show remarkable promise in preclinical studies, but their clinical efficacy has been more modest. Currently, the forthcoming focus in the field of drug development is anticipated to be on the development of inhibitors which is founded on a multitarget approach. In addition, different combination strategies are being pursued in clinical trials, including the combination of epigenetic therapies with targeted therapies and immunotherapy. Given the fundamental roles of dysregulated KMTs and KDMs in AD development, these inhibitors may hold the key to regulating AD-related pathologic changes by inhibiting the main molecular pathways and emerge as an effective and promising strategy for AD therapy.

## 3. Energy metabolism and AD

Energy metabolism is the process of releasing, transferring, storing, and utilizing energy as part of the material metabolism process, in which the mitochondria play an important role. Dysfunctional energy metabolism in the brain is considered a precipitating factor and a central link in many neurodegenerative diseases such as AD. It has been shown that disorders of energy metabolism occur in the central nervous system and periphery early in AD concurrent with the development of AD pathology. These disruptions include mitochondrial dysfunction and glucose, lipid, and amino acid metabolism disorders. The tricarboxylic acid cycle (TCA cycle), also known as the Krebs cycle, is the final metabolic pathway of the three major nutrients and the pivot of the three major metabolic processes (Fig. 2). Conversely, pathological changes in AD can affect energy metabolism homeostasis. Oxidative stress induced by Aβ42 oligomers, for example, may impair glucose metabolism [57]. Consequently, there is a complex interplay between energy metabolism and AD.

### 3.1 Glucose metabolism and AD

Glucose is the primary source of energy in the brain. Because it cannot be synthesized or stored in the central nervous system, glucose must enter the brain from the periphery through the blood-brain barrier (BBB) with the help of sodium-dependent glucose transporter 1/2 and glucose transporters (GLUTs) [58, 59].

### 3.1.1 Decreased glucose intake

Several positron emission tomography studies have shown that the entorhinal cortex and parietal lobes have a deficit in glucose uptake in mild cognitive impairment, suggesting that impaired glucose metabolism plays a key role in the early pathogenesis of AD [60]. Glucose deprivation has been shown to increase the expression of the unfolded protein response, which facilitated tau protein phosphorylation in vivo and *in vitro* models [61]. Besides, the experiment using the postmortem brain samples from AD patients and age-matched non-AD patients demonstrated that impaired glucose metabolism inhibited nonamyloidogenic α-secretase processing of APP via the peroxisome proliferator activated receptor gamma coactivator 1 alpha/forkhead box protein O3a (PGC-1α/FoxO3a) pathway, thus promoting Aβ generation [62]. The increase in glucose uptake was found to boost memory by changing the distinctive chromatin acetylation status of the promoter regions of BDNF and fibroblast growth factor 1 in neuronal cells, astrocytes, and microglial cells of the murine hippocampus [63]. Moreover, a study conducted in both cell culture and animals showed that high glucose levels led to neuronal mitochondrial dysfunction and resultant insulin resistance in an adenosine monophosphate-activated protein kinase-dependent manner [64]. However, these regulatory mechanisms have not been observed in AD. In the brain, GLUT1 is expressed mainly in glia and endothelial cells [65]. Reduced GLUT1 expression in BBB worsens cerebrovascular degeneration, neuropathology, and cognitive function in AD [66]. In addition, glycogen synthase kinase-3β (GSK-3β) is a highly conserved multifunctional serine/threonine protein kinase whose activity is negatively regulated by protein kinase B (PKB/AKT). The advanced glycation end products (AGEs) play a key role in AD [67]. They were discovered to cause spatial memory deficit and tau protein hyperphosphorylation through receptor for advanced glycation end products (RAGE)-mediated AKT/GSK-3β pathway in the human neuroblastoma SK-N-SH cells, hippocampal neuron, and the rat brain [68].

### 3.1.2 Insulin resistance

Phosphatidylinositol 3-kinase (PI3K), an intracellular phosphatidylinositol kinase with serine/threonine kinase and phosphatidylinositol kinase activities, is involved in the regulation of various cellular functions such as glucose transport. It has been shown that insulin binds to its receptor and is essential for the metabolism of Aβ and tau protein via the PI3K/AKT signaling pathway [69]. Insulin was also found to promote the expression of NEP and IDE in astrocytes through the activation of extracellular signal-regulated kinase (ERK)-mediated pathway [70]. However, Steen et al. [71] reported that impaired insulin signaling caused by aberrant fluctuations in blood glucose levels resulted in dephosphorylation of GSK-3, which in turn encouraged the Aβ deposition and tau protein phosphorylation in the postmortem brain tissues of AD and control cases. Additionally, a study conducted by Ho et al. [72] in AD mouse model demonstrated that insulin resistance affected the production and clearance of Aβ by taking effect on γ-secretase and IDE.

### 3.1.3 O-GlcNAcylation

Phosphorylated and O-GlcNAcylated tau proteins in the brain interact to promote the formation of NFTs. Impaired cerebral glucose metabolism due to the absence of GLUT1/3 can decrease tau protein glycosylation, while increasing tau protein phosphorylation [73]. O-GlcNAcase insufficiency can elevate the level of O-GlcNAcylation, which inhibits the activation of necroptosis factors and the excessive activation of astrocytes and microglia, ultimately ameliorating neuronal death and neuroinflammation in AD. Furthermore, increased O-GlcNAc level is associated with restored mitochondrial function, normal mitochondrial morphology, and reduced Aβ deposition [74], but the detailed mechanisms are still unclear.

### 3.1.4 Ketone body metabolism

Ketone bodies, a generic term for acetoacetic acid, β-hydroxybutyric acid (βOHB), and acetone, are metabolized more rapidly than glucose and can bypass glycolysis to enter directly into the TCA cycle. A large number of studies have shown that ketone bodies can reduce AD pathologies including extracellular Aβ plagues, NFTs, and neuronal loss through various pathways [75]. Ketone bodies can promote Aβ_1-40_ transendothelial transport via enhancing the function of lipoprotein receptor-related protein 1 (LRP1) and P-glycoprotein transporter and phosphatidylinositol-binding clathrin assembly protein (PICALM), which are responsible for Aβ clearance [76]. It has been found that βOHB increased the level of NEP via G protein-coupled receptor 109A in the five familial AD mutations mice and HT22 cells [77] and prevented the increase in the expression of BACE1 caused by GLUT inhibitor in SH-SY5Y cells [78]. βOHB was also discovered to reduce Aβ plague formation and microgliosis in the five familial AD mutations mice by inhibiting nod-like receptor pyrin domain containing 3 (NLRP3) inflammasome activation [79]. In addition, a recent study has demonstrated that βOHB ameliorated the downregulation of tyrosine kinase receptor A (TrkA) expression in Aβ-treated SH-SY5Y cells by inhibiting histone deacetylase 1/3 (HDAC1/3) [80]. In APP/PS1/Tau transgenic mice, circulating βOHB concentrations increased by alternative-day-fasting have been shown to improve the expression of PSD95 and synapsin and reduce Aβ oligomers as well as tau hyperphosphorylation [81].

### 3.2 Lipid metabolism and AD

Most solid components of the brain consist of lipids, including triglycerides, fatty acids, phospholipids, sterol lipids, and sphingolipids. These lipids are important for maintaining BBB function and are involved in oxidative stress and energy balance. Studies have shown that increases in sphingolipids and triglycerides are associated with Aβ production. The increase of ceramides, a kind of sphingolipids, can promote lipid peroxidation, oxidative stress, mitochondrial dysfunction, and neuronal death. Additionally, the decreased levels of specialized pro-resolution mediators from eicosapentaenoic acid and docosahexaenoic acid and the increased production of prostaglandin E from arachidonic acid can promote inflammation, and thus increasing the risk of AD [82].

Genome-wide association studies have identified some AD-related genes involved in lipid trafficking or metabolism, such as APOE, clusterin/apolipoprotein J, triggering receptor expressed on myeloid cells 2 (TREM2), PICALM, sterol regulatory-element binding protein 2 (SREBP2), and the ATP-binding cassette subfamily A (ABCA) [83].

### 3.2.1. APOE4

APOE is a polymorphic protein that regulates lipoproteins conversion and metabolism. APOE4, an isoform of APOE, is considered the most significant risk factor for late-onset AD. Numerous studies have shown that APOE4 exerts important roles in Aβ metabolism, tau protein phosphorylation, neuroinflammation, synaptic plasticity, and the functions of BBB and mitochondria. APOE4 was shown to enhance APP transcription and Aβ synthesis by activating the non-canonical mitogen-activated protein (MAP)-kinase cascade involving dual leucine-zipper kinase (DLK), mitogen-activated protein kinase kinase 7 (MKK7), and ERK1/2 *in vivo* and *in vitro* [84]. LRP1, a key receptor for Aβ clearance at the BBB, can regulate the Aβ clearance by binding to APOE and PICALM [85, 86]. Conversely, Wan et al. [87] discovered that Aβ stimulated lipolysis in a manner that appeared partially dependent on the PKA/ERK signaling pathway and induced leptin and IL-6 secretion in cultured human adipose tissue. This process leads to the release of fatty acids, triggering lipid deposition and insulin resistance, consequently affecting lipid degradation.

APOE4 was also found to induce the phosphorylation of tau proteins at certain sites through the activation of calpain-dependent kinase 5 (CDK5) signaling pathway in AD mouse model [88]. The present study has provided compelling evidence that the APOE epsilon4 affected the immune system through down-regulation of the IL-7/IL-7R signaling pathway, contributing to neuroinflammation in AD patients [89]. In terms of morphology and function of synapses, induced pluripotent stem cells-derived cerebral organoids from AD patients carrying APOE epsilon4/epsilon4 have been shown a decrease in synaptic integrity [90]. APOE4 also impairs hippocampal LTP through reducing the phosphorylation of cAMP response element binding protein (CREB) as well as Ca^2+^/calmodulin-dependent protein kinase IIα (CaMKIIα) [91]. Another study using *in vivo* models discovered that APOE4 led to fewer dendritic spine numbers by elevating the activity of calcium/calmodulin-dependent protein phosphatase 2B (PP2B) [92]. However, the exact mechanisms are unclear. Besides, Yin et al. [93] found that the PGC-1α-sirtuin3 signaling pathway mediated synaptic damage by APOE4, ultimately resulting in cognitive impairment *in vivo* and *in vitro* models. It has been demonstrated that humanized APOE4 mice showed a leaky BBB, increased matrix metallopeptidase 9, impaired tight junctions, and reduced astrocyte end-foot coverage of blood vessels [94]. APOE epsilon4 carriers were found to have lower expression levels of mitochondrial fusion- and division-related proteins compared to non-carriers [95].

### 3.2.2. TREM2, SREBP2, and ABCA family

TREM2, located on the surface of microglia, can affect the metabolism of cholesterol, myelin, and phospholipids. TREM2 can increase the degradation of amyloid plaques by promoting microglial phagocytosis [96], and act as a ligand for phospholipids, lipoproteins, and apolipoproteins [97]. Conversely, a study using AD mice found that TREM2 knockdown reduced microglia activation and aggregation around Aβ plagues, which increased Aβ plague pathology [98]. Deficiency of TREM2 was demonstrated to impair the microglial response to tau accumulation in the brain of hTau and PS19 mice [99]. Besides, Zhong et al. [100] reported that TREM2 suppressed the activation of classical complement cascade and complement-mediated synaptic loss in AD mice models by directly binding to C1q, the initiator of the classical complement pathway.

SREBP2, a sterol regulatory transcription factor, has an essential role in regulating cholesterol and fatty acid homeostasis. Overexpression of SREBP2 accelerates oxidative damage, Aβ accumulation, neuroinflammation, and cognitive decline [101]. Specific knockdown of SREBP2 in astrocytes was discovered to significantly decrease Aβ and tau protein levels [102]. On the contrary, oligomeric Aβ_42_ inhibited SREBP2 activation through AKT inhibition [103].

The ABCA transporters family plays a central role in the maintenance of cholesterol-homeostasis in the brain and has a prominent role in AD development. These transporters have been reported to target the primary hallmark of AD pathogenesis, namely, the Aβ hypothesis. Among the ABCA transporters family, ABCA1, 2, 5, and 7 are all associated with Aβ metabolism. It has been found that the increased ABCA1 led to an increase in lipidation of APOE. The lipidated APOE was discovered to facilitate the proteolytic degradation of Aβ by NEP and IDE and promote the clearance of Aβ in the hippocampus of male Wistar rats [104]. It has been reported that ABCA2 was associated with β/γ-secretase, which hydrolyzed APP in the N2a mouse neuroblastoma cells [105]. Another study showed that the overexpression of ABCA2 increased low-density lipoprotein receptor density, which decreased Aβ deposition *in vivo* [106]. ABCA5 can reduce Aβ production but does not promote Aβ clearance [107]. In ABCA7 haplodeficient microglia, Aikawa et al. [108] reported that proper endosomal-lysosomal trafficking might be disturbed, resulting in abnormal accumulation of Aβ. In addition, deletion of ABCA7 facilitated the processing of APP to Aβ through increasing the levels of BACE1 and SREBP2 in primary neurons and mouse brains. This study also demonstrated that knockdown of ABCA7 expression in neurons caused endoplasmic reticulum stress highlighted by increased levels of protein kinase R-like endoplasmic reticulum kinase (PERK) and increased phosphorylation of eukaryotic initiation factor 2α (eIF2α), resulting in cognitive decline [109]. Apart from targeting the Aβ hypothesis, lacking ABCA1 was found to result in an impaired hippocampal neurite morphology in APP/PS1 mice [110]. In summary, lipid substances and related regulators of lipid metabolism may be involved in the pathogenesis of AD by interacting with crucial mechanisms involved in the etiology of AD.

### 3.3. Amino acid metabolism and AD

As neurotransmitters in the central nervous system, amino acids are involved in the synthesis and metabolism of various active substances in the body and play important roles in learning, memory neurotransmission, and energy metabolism. Disorders of amino acid metabolism have also been reported in patients with AD [111].

### 3.3.1. Glutamate, γ-aminobutyric acid, glutamine, and branched-chain amino acids

Excitatory amino acids such as glutamate (Glu) have excitatory effects on neurons. Their release can damage neuronal cells and lead to other pathologies, including learning and memory impairment. Inhibitory amino acids, like γ-aminobutyric acid (GABA) have postsynaptic inhibitory effects and can protect neurons from damage.

Glutamine (Gln) is widely distributed in the brain and is the most abundant amino acid in cerebrospinal fluid. It plays a significant role in lipid and protein synthesis, nucleic acid synthesis, and autophagy [112]. The level of Gln in AD is decreased and is proven to have protective effects on AD [113]. For instance, Gln was found to protect against oxidative stress-induced injury *in vitro* and *in vivo* through activating the Wnt3a/β-catenin signaling pathway [114]. Besides, Gln is an important precursor of Glu and GABA. Following its entry into the cell through the transporter protein, Gln generates Glu under GLS or GLS2, followed by α-KG and GABA under the catalytic action of GLUD and GAD respectively. Under normal physiological conditions, Glu and GABA maintain the balance and normal function of the nervous system, but both are significantly decreased in AD [115, 116]. GABA reacts with α-KG to generate SSA and, ultimately, succinate, which constitutes the GABA shunt. The GABA shunt, a bypass of the TCA cycle, provides approximately 20% of the energy to the brain, thereby altering AD pathology to some extent. It has been documented that Glu promoted the polarization of M2 macrophage and reduced the inflammatory response through the α-KG-JMJD3 pathway [117]. Succinate is generated from Gln via GABA bypass. It has been demonstrated to stabilize hypoxia-inducible factor-1α (HIF-1α) by inhibiting prolyl hydroxylase or enhancing ROS enzyme activity, thereby promoting the release of inflammatory factors including IL-1β [118, 119]. However, these regulatory mechanisms have not yet been reported in AD. Considering that HIF-1α is associated with multiple pathological features of AD and microglia is an important macrophage in the brain, it is worth to investigate whether Gln in microglia can also influence the release of pro- and anti-inflammatory factors via the α-KG-JMJD3 or HIF-1α pathway to delay the development of AD.

Branched-chain amino acids (BCAAs) include leucine, isoleucine, and valine. The level of BCAAs is elevated in AD. A recent study has found that the supplementation of BCAAs increased phosphorylated tau in HT22 hippocampal neurons. The restriction of BCAAs effectively lowered amyloid and tau pathology in APP/PS1 mice. This study also reported that the application of BT2, a BCAAs-lowering compound, reduced Aβ_42_ and enhanced cortical and hippocampal neurotransmitter levels in five familial AD mutations mice [120]. However, the specific mechanisms by which BCAAs affect AD pathology need to be further studied. Additionally, BCAAs, especially leucine, play critical roles in the brain as key nitrogen donors for Glu synthesis [121], and Glu is a precursor of GABA. Consequently, disturbances in BCAAs levels may lead to a serious imbalance in these key neurotransmitters.

### 3.3.2. Serine and tryptophan

L-serine is a non-essential amino acid and is produced mainly in glial cells, especially astrocytes. L-serine is the obligatory precursor of D-serine, a co-agonist of synaptic NMDARs required for synaptic plasticity in the hippocampus. L-serine is approved by FDA for supplemental use, while D-serine is not. It has been found that impaired synaptic plasticity and memory in triple transgenic AD mice were rescued by the dietary supplement of L-serine [122]. A recent study reported that D-serine levels were elevated in the serum and cerebrospinal fluid of AD patients compared with controls [123] and Aβ oligomers might lead to elevated D-serine levels by upregulating serine racemase in cultured hippocampal neurons as well as APP/PS1 transgenic mice [124]. Reducing D-serine in the brain was shown to partially attenuate neuronal loss and reactive astrogliosis in AD mouse model [125]. However, Juliette et al. [122] discovered that the level of D-serine in the hippocampus was lower in AD mice than in control mice and D-serine supplement was found to rescue the spatial memory deficits. Another study showed that the concentrations of D-serine in the serum and cerebrospinal fluid were unaltered in AD patients [126]. It can be seen that the expression of D-serine in AD is controversial, and further studies are needed in the future to clarify the regulatory mechanisms of D-serine in AD progression.

Tryptophan is an essential amino acid whose metabolism is carried majorly through the kynurenine pathway. The kynurenine pathway is intricately linked to AD pathogenesis owing to the influence of kynurenine metabolites on amyloid aggregation, modulation of neuroinflammation, oxidative stress, and so on [127-129]. Recently, bioinformatics analysis identified five tryptophan metabolism-related genes associated with AD, including propionyl-CoA carboxylase subunit beta (PCCB), TEA domain transcription factor 1 (TEAD1), phenylalanyl-tRNA synthetase subunit beta (FARSB), neurofascin (NFASC), and ezrin (EZR). The expressions of PCCB and NFASC were down-regulated in the hippocampus of APP/PS1 mice [130], but the exact mechanisms have not yet been clarified.

In summary, glucose, lipids, and proteins, the three primary energy-providing components, play critical roles in the pathogenesis of AD (Table 4).

**Table 4:** Energy metabolism and AD.

Energy metabolism	Direction (→/←)	Affected pathways/genes/proteins	Pathologies/phenotypes in AD	Experimental subjects	References
**Glucose metabolism**	→	Glucose deprivation →Unfolded protein response↑	Tau protein phosphorylation↑	Hamsters, cells	[61]
	→	Impaired glucose metabolism→PGC-1α↓→FoxO3a↑→APP processing (α- secretase) ↓	Aβ generation↑	Human brain tissues, cells	[62]
	→	AGEs→ RAGE- mediated AKT/GSK-3β pathway	Spatial memory deficit↑; Tau protein hyperphosphorylation↑	Mice, cells	[68]
	→	Impaired insulin signaling→ GSK3 dephosphorylation	Aβ deposition and tau protein phosphorylation↑	Human brain tissues	[71]
	→	Insulin →activation of ERK- mediated pathway →NEP, IDE↑; Insulin resistance →γ-secretase↑, IDE↓	Aβ production↑ and Aβ clearance↑	Cells, mice	[70, 72]
	→	Lack of GLUT1/3→Tau protein glycosylation↓→Tau protein phosphorylation↑	NFT↑	Human brain tissues, mice, and cells	[73]
	→	O-GlcNAcase insufficiency→O-GlcNAcylation↑→Necrotrophic apoptotic factor↓ and excessive activation of astrocytes and microglia	Neuronal death and neuroinflammation in the brain↓; Mitochondrial function↑, normal mitochondrial morphology↑, and Aβ deposition↓	Human brain tissues, mice, and cells	[74]
	→	Ketone bodies→LRP1, P-glycoprotein transporter and PICALM↑→Aβ_1-40_ transendothelial transport↑	Aβ clearance↑	Cells	[76]
	→	①βOHB→G protein-coupled receptor 109A→NEP↑; ②βOHB→BACE1↓; ①βOHB→NLRP3 inflammasome activation↓; ④βOHB→HDAC1/3↓→TrkA↑; ⑤βOHB↑ (increased by alternative-day-fasting) →PSD95↑, synapsin↑, Aβ oligomers↓, tau hyperphosphorylation↓	①Aβ degradation↑; ②Aβ generation↓; ①Aβ plague formation, microgliosis↓; ④Aβ-induced neurotoxicity↓; ⑤Synaptic plasticity↑, Aβ oligomers↓, tau hyperphosphorylation↓	①Cells; ②Mice; Cells; ①Human samples, mice, and cells; ④Cells; ⑤Mice	[77-81]
	←	Aβ_42_→Oxidative stress → Impaired glucose metabolism → Synaptic dysfunction and neuronal death	Aβ_42_↑; Synaptic dysfunction and neuronal death↑	Human brain tissues	[57]
**Lipid metabolism**	→	APOE4→Non-canonical DLK→MKK7→ERK1/2 MAP-kinase cascade →APP↑	Aβ synthesis↑	Mice, cells	[84]
	→	LRP1+APOE; LRP1+PICALM	Aβ clearance↑	Human brain tissues, mice, and cells	[85, 86]
	→	APOE4→Activation of CDK5 signaling pathway	Tau protein phosphorylation↑	Mice	[88]
	→	APOE4→IL-7/IL-7R signaling pathway↓	Neuroinflammation↑	Patients	[89]
	→	①APOE4→ Phosphorylation of CREB and CaMKⅡα↓→Hippocampal LTP↓; ②APOE4→PP2B↑→Dendritic spine numbers↓; ①APOE4→PGC-1α-Sirtuin3 signaling pathway→ Synaptic damage↑	Synaptic function↓, cognitive dysfunction↑	①Mice; ②Mice; ①Mice, cells	[93, 95, 250, 251]
	→	①TREM2→Microglial phagocytosis↑→Aβ degradation↑; ②TREM2 knockdown→ Microglia activation and aggregation around Aβ plagues↓; ①TREM2 deficiency→ The microglial response to tau accumulation↓; ④TREM2→ The activation of classical complement cascade and complement-mediated synaptic loss↓	①Aβ degradation↑; ②Aβ plague↑; ①Tau accumulation↓; ④Synaptic function↓	①Mice, cells; ②Mice, cells; ①Mice; ④Human brain tissues, mice	[96, 98-100]
	→	Overexpression of SREBP2; Knockdown of SREBP2	Oxidative damage, Aβ accumulation, neuroinflammation, and cognitive decline↑; Aβ and tau protein↓	Mice, cells	[101, 102]
	←	Oligomeric Aβ_42_→AKT inhibition→SREBP2 activation↓	Oligomeric Aβ_42_	Mice, cells	[103]
	→	①ABCA1→Lipidation of APOE→ Proteolytic degradation of Aβ by NEP, IDE; ②ABCA2→β/γ-secretase; ①ABCA2 overexpression →Low-density lipoprotein receptor density↑; ④ABCA5 →Aβ production↓; ⑤ABCA7 knockout→Endosomal-lysosomal trafficking; ⑥ABCA7 deletion→SREBP2, BACE1↑; ABCA7 knockdown→ Endoplasmic reticulum stress→PERK-eIF2α pathway	Aβ clearance↑; Aβ production↓; Aβ deposition↑; Aβ accumulation↑; Cognitive function	①Rats; ②Cells; ①Mice; ④Human brain tissues, mice, cells; ⑤Mice, cells; ⑥Mice, cells; Mice, cells	[104-109]
	→	ABCA1↓→impaired hippocampal neurite morphology	Cognitive function↓	Mice	[110]
	←	Aβ→PKA/ERK signaling pathway →Lipolysis; Aβ→Leptin and IL-6 secretion	Aβ↑	Cells	[87]
Amino acid metabolism	→	Gln→Wnt3a/β-catenin signaling pathway↑→SOD↑, glutathione peroxidase↑, malondialdehyde↓	Oxidative stress-induced injury↓	Mice, cells	[114]
	→	BCAAs↓ →Amyloid and tau pathology↓, cortical and hippocampal neurotransmitter levels↑	Aβ↓; Tau phosphorylation↓; Cognitive function↑	Mice, cells	[120]
	→	L-serine↑→Stimulates the NMDAR	Synaptic plasticity and memory↑	Human brain tissues, mice	[122]
	→	D-serine↓ →Neuronal loss and reactive astrogliosis↓; D-serine supplement →Spatial memory deficits↓	Neuronal loss and reactive astrogliosis↓; Spatial memory deficits↓	Mice; Human brain tissues, mice	[122, 125]
	→	Tryptophan →Kynurenine pathway (kynurenine metabolites)	Aβ aggregation; Modulation of neuroinflammation; Oxidative stress	Elderly individuals; Human brain tissues, cells; Rats	[127-129]
	→	Tryptophan metabolism-related genes (PCCB, TEAD1, FARSB, NFASC, EZR)	None	Mice	[130]
	←	Aβ oligomers→ serine racemase↑→D-serine↑	Aβ oligomers↑	Mice, cells	[124]

Note: “→” denotes the role of energy metabolism on AD; “←” denotes the role of AD on energy metabolism; “↑” denotes increased levels; “↓” denotes reduced levels.

### 3.4 Mitochondrial dysfunction

Mitochondria are the major organelles involved in energy metabolism, where metabolic activities, such as glucolipid metabolism, amino acid metabolism, and the TCA cycle interact with each other. Mitochondrial biosynthesis, dynamics, autophagy, and ROS production mediated by apoptosis affect the maintenance of mitochondrial homeostasis. In addition, the mitochondrial cascade hypothesis proposes that mitochondrial dysfunction triggers AD pathology and may lead to neurodegenerative lesions, such as Aβ deposition, NFTs, synaptic loss, and axonal transport defects.

Mitochondrial homeostasis is achieved through continuous division and fusion. In AD, inhibition of dynamin-related protein 1 is associated with loss of neuronal synapse and dendritic spine, defection of axonal transport, Aβ deposition, and tau protein hyperphosphorylation [131, 132]. Overexpression of mitochondrial fusion proteins including optic atrophy 1 was demonstrated to alleviate the overproduction of ROS in the mitochondria of AD brains [133]. On the contrary, Aβ deposition and tau hyperphosphorylation affect mitochondria, leading to mitochondrial fragmentation and interfering with energy metabolism [134, 135]. In addition to kinetic abnormalities, mitochondrial biosynthesis and mitophagy were impaired in human AD brain samples, AD-induced pluripotent stem cell-derived neurons, and transgenic animal models of AD. Defects in mitophagy induce the accumulation of dysfunctional mitochondria, thereby promoting AD pathology [136].

Essential enzymes and intermediate metabolites of the TCA cycle are closely associated with AD development. The activities of key enzymes, such as IDH, SDH, and pyruvate dehydrogenase, are significantly decreased in AD, affecting ATP production and energy metabolism [137]. Moreover, the antioxidant function of the organism gradually decreases with age, resulting in excessive production of ROS, which attacks ACO2 during isocitric acid synthesis, thus affecting mitochondrial function [138]. Intermediate metabolites enter the cytoplasm via carriers on the inner mitochondrial membrane. These metabolites participate in mitochondria-to-nucleus retrograde signaling, which regulates AD by influencing mitochondrial homeostasis.

## 4. Energy metabolism and histone methylation

Of note, multiple studies have reported crosstalk between energy metabolism and histone methylation.

### 4.1 Energy metabolism affects histone methylation

In energy metabolism, particularly in the TCA cycle, FAD acts as a coenzyme for the conversion of succinate to fumarate. LSD1 is a FAD-dependent demethylase that catalyzes the demethylation of H3K4me1/2 and H3K9me1/2 [139]. The catalytic activity of LSD1 may be directly related to the cellular metabolic state via fluctuations in the FAD/FADH2 (the reduced form of FAD) ratio, depending on FAD oxidation processes such as the TCA cycle [140]. Moreover, as a crucial intermediate in the TCA cycle, α-KG not only plays a role in DNA demethylation but also serves as a co-factor in histone demethylases of the JMJD family. This intermediate can be supplied directly or indirectly by mitochondria and affects the epigenetic regulation of the nuclear genome. When mitochondrial metabolism is disturbed, α-KG decreases histone methylation, whereas succinate and fumarate may exert opposite effects [141]. Obesity, caused by lipid metabolism disorders, is one of the main causes of AD pathogenesis. Adipose tissues include white adipose tissue, brown adipose tissue, and beige adipose tissue. Promoting the thermogenic function of brown adipose tissue and browning of beige adipose tissue in white adipose tissue can reduce obesity. Peroxisome proliferator-activated receptor γ (PPARγ) belongs to one of the intranuclear hormone receptor superfamily members. PPARγ and other receptors from this family such as PPARα, play important roles in adipocyte production and energy metabolisms. There is evidence that uncoupling protein 1 (UCP1) is the center of brown adipose tissue thermogenesis and systemic energy homeostasis. It was found that 1% α-KG dissolved in drinking water promoted the formation of beige fat and prevented obesity in middle-aged mice [142]. In addition, IDH1 is an essential metabolic enzyme that catalyzes the conversion of isocitric acid to α-KG. Chromatin immunoprecipitation analysis revealed that IDH1-mediated increase of α-KG reduced the level of H3K4me3 in the promoters of brown adipogenic genes, including PPARγ and UCP1, which was accompanied by their decreased expression *in vivo* and *in vitro*, respectively [143]. However, whether α-KG may affect AD-like pathologies by regulating the expression of genes related to brown adipogenesis, such as PPARγ and UCP1, needs to be further explored.

In addition to being influenced by related enzyme activities and intermediate metabolites, histone methylation is affected by changes in SAM levels. SAM is produced through the coupling of folate and methionine cycles in the cytoplasm, enters the nucleus as a methyl source for histones, and participates in histone methylation. Thus, SAM levels are regulated by methionine. When methionine levels are deficient, SAM levels decrease, which subsequently affects the transcription of genes related to Aβ processing, nerve growth factor, and epigenetic modifications [144]. Additionally, mitochondrial bifunctional enzymes can use methylenetetrahydrofolate to synthesize formyltetra-hydrofolate, while consuming one-carbon groups during purine biosynthesis. When mitochondrial bifunctional enzymes are not expressed during purine biosynthesis, the consumption of one-carbon groups is decreased. Accordingly, the synthesis of serine increases, whereas excess serine in the cytoplasm generates cysteine, which reacts with ATP to synthesize SAM, ultimately increasing histone methylation levels [145].

Studies have found that βOHB induced a decrease in the H3K27me3 as well as an increase in the H3K4me3 occupied in BDNF promoters and both were dependent on the activation of cAMP/PKA signaling pathways in the primary hippocampal neurons and the murine hippocampal neuronal cell line HT22 [146, 147]. PR/SET domain containing protein 9 (PRDM9) is a histone methyltransferase that can tri-methylate H3K4 and H3K36 at surrounding nucleosomes. Gln was demonstrated to promote transcriptional induction of brown adipogenic and thermogenic genes, such as PPARγ and UCP1, through C/EBPβ-PRDM9-mediated H3K4me3 *in vivo* and in vitro models [148].

### 4.2 Histone methylation affects energy metabolism

SETD2 is a methyltransferase that regulates H3K36me3 levels. A recent study reported that SETD2 deficiency inhibited the expression of cholesterol efflux genes such as ABCA1, resulting in lipid accumulation *in vivo* and *in vitro* [144]. It has been shown that PPARγ can be inhibited by G9a and EZH2 and activated by MLL3/4 [149-151]. Another study has shown that JHDM2A played an essential role in directly regulating PPARα and UCP1 *in vivo* and *in vitro* experiments, two of the important genes involved in controlling energy balance [152]. LSD1 mediates demethylation of H3K9me1 and H3K9me2. Sambeat et al. [153] observed that LSD1 was recruited to the UCP1 promoter by directly interacting with zinc finger protein 516 for transcriptional activation by H3K9 demethylation at the UCP1 promoter region, which promoted the function of thermogenic adipose tissue. The knockdown of PHF2 was found to result in a reduction in the expression of major genes related to fat cell differentiation, including PPARγ and CCAAT/enhancer binding protein α (C/EBPα) *in vitro* [154]. PR domain-containing 16 (PRDM16) is a dominant activator of the biogenesis of beige adipocytes and an attractive target for improving metabolic health. Wang et al. [155] found that EHMT1 stabilized PRDM16 protein by competing with amyloid protein-binding protein 2 for direct binding to PRDM16 and blocking PRDM16 polyubiquitination, thereby promoting beige adipocyte biogenesis.

The administrations of PPARγ and PPARα agonists can decrease amyloid levels, reduce inflammation, and ameliorate synaptic function [156-159]. It has been found that the deletion of UCP1 in Tg2576 mice increased body temperature, which elevated Aβ and tau levels and decreased synaptic protein levels [160]. However, whether PPARα, PPARγ, and UCP1 mediate the regulation of AD-related pathologies by G9a, EZH2, MLL3/4, JHDM2A, LSD1, PHF2, and EHMT1 need to be further explored.

SREBP1 and SREBP2 are responsible for regulating fatty acid and cholesterol synthesis, respectively [161]. In vivo and *in vitro* models, inhibition of DOT1L was found to lead to suppressed SREBP1/2 lipid synthetic pathways that consequently enhanced macrophage inflammatory activation [162]. A recent study has demonstrated that SETD8 modulated SREBP1 by mono-methylation of H4K20 to promote the lipogenesis of renal cell carcinoma [163]. Tang et al. [164] observed that UTX promoted prostate tumorigenesis and lipid metabolism by binding to the promoter of SREBP1c, one of the isoforms of SREBP1, to increase SREBP1c transcription *in vivo* and *in vitro* experiments. Besides, ligand-activated liver X receptors (LXRs) are transcription factors that play an essential role in promoting lipogenesis. The activation of LXRα, one of the subtypes of LXRs, was discovered to increase JMJD2B recruitment to LXR response elements and reduce H3K9me2/3 enrichment in the promoter of LXRα-dependent lipogenic genes such as SREBP1 [165]. The relationship between SREBP2 and AD has been explained previously, and the knockdown of SREBP1 has been reported to reduce the palmitate-induced increase in BACE1 expression and subsequent Aβ genesis in the mouse hippocampus and mouse Neuro-2a neuroblastoma cells [166]. However, it remains unknown whether DOT1L, SETD8, UTX, and JMJD2B affect AD-related pathologies via SREBP1 or SREBP2.

**Table 5:** Energy metabolism and histone methylation.

Energy metabolism	Direction (→/←)	KMTs/KDMs	Experimental subjects	References
**FAD**	→	LSD1 (FAD-dependent demethylase) demethylation of H3K4me1/2 and H3K9me1/2	None	[139]
**α-KG**	→	JMJD family (α-KG-dependent demethylase)	None	[141]
**IDH1**	→	H3K4me3 level in the promoters of PPARγ and UCP1↓	Mice, cells	[143]
**SAM**	→	Methyl donor for HMTs	None	[145]
**βOHB**	→	cAMP/PKA signaling pathways→BDNF promoters (H3K27me3↓, H3K4me3↑)	Mice, cells	[146, 147]
**Gln**	→	C/EBPβ/PRDM9 pathways→Brown adipogenic and thermogenic genes↑	Human tissues, mice, and cells	[148]
**ABCA1↓→lipid accumulation**	←	SETD2 deficiency	Mice, cells	[144]
**PPARγ**	←	Inhibited by EHMT2 (G9a) and EZH2; Activated by MLL3/4	Cells; Mice, cells; Mice	[149-151]
**PPARα and UCP1↑**	←	JHDM2A	Mice, cells	[152]
**UCP1 promoter (H3K9 demethylation)**	←	LSD1+ zinc finger protein 516	Mice, cells	[153]
**PPARγ, C/EBPα↓**	←	PHF2 knockdown	Cells	[154]
**Beige adipocyte biogenesis↑**	←	EHMT1 (stabilizes PRDM16)	Mice, cells	[155]
**①SREBP1/2 lipid synthetic pathways↓; ②SREBP1 (H4K20me); ①SREBP1c**	←	①DOT1L; ②SETD8; ①UTX	①Mice, cells; ②Human tissues, cells; ①Mice, cells	[162-164]
**SREBP1 (H3K9me2/3)**	←	JMJD2B (recruited by LXRα)	Mice, cells	[165]
**MafA, Slc2a2, and Ins1/2↑**	←	SET7/9 (recruited by pancreatic duodenal homeobox 1)	Mice, cells	[167, 168]
**Mitochondrial glucose and lipid metabolism disorder and oxidative stress (Sirtuin 3 expression↓ (H3K4me3) or AMP-activated protein kinase/Sirtuin 3 pathways↓)**	←	KDM5B	Mice, cells	[173]

Note: “→” denotes the role of energy metabolism on AD; “←” denotes the role of AD on energy metabolism; “↑” denotes increased levels; “↓” denotes reduced levels.

In addition, pancreatic duodenal homeobox 1, an essential transcription factor for pancreatic differentiation and development, can promote the expression of insulin, glucokinase, and other important genes in pancreatic β-cells. Studies have shown that pancreatic duodenal homeobox 1 recruited SET7/9, which methylated H3K4 in the promoter region of insulin secretion-related genes, such as β-cell-specific transcription factor MafA, the glucose transporter Glut2 (Slc2a2), and preproinsulin (Ins1/2) [167, 168]. Insulin can cross the BBB, bind to the insulin receptor (IR) which are mainly located in the olfactory bulbs, hypothalamus, cerebral cortex, and hippocampus [169], and lead to IR autophosphorylation. The phosphorylated IR then phosphorylates the insulin receptor substrate. The activated insulin receptor substrate not only regulates glucose metabolism, but also inhibits GSK-3α and GSK-3β to regulate APP expression, Aβ clearance, and tau protein phosphorylation levels through the PI3K/AKT pathway [170-172]. AMP-activated protein kinase is a vital energy sensor, and its activation comes with metabolic benefits. Sirtuin 3 plays an important role in maintaining mitochondrial function and energy metabolism. A previous study has found that the overexpression of KDM5B triggered mitochondrial glucose and lipid metabolism disorder and oxidative stress via transcriptional inhibition of sirtuin 3 expression by demethylating H3K4me3 or inactivation of AMP-activated protein kinase/sirtuin 3 pathways in the models of diabetic peripheral neuropathy model [173]. Diabetic peripheral neuropathy is one of the most common diabetes-related complications and diabetes is proven to be a risk factor for the development of AD, but whether this regulatory mechanism exists in AD needs further explored.

In conclusion, a complex connection exists between energy metabolism and histone methylation (Table 5), both of which are critical for the development of AD. However, the underlying mechanisms remain unknown. Therefore, understanding the relationship between energy metabolism and histone methylation is important for AD.

## 5. Discussion

AD is a chronic neurodegenerative disorder, and its complexity makes its cure an unsolved problem for the time being. Currently, as for drugs for AD treatment, anticholinesterase inhibitors, N-methyl-D-aspartate receptor antagonists [174], and antibodies against Aβ such as aducanumab and lecanemab [175], have been approved by FDA. However, there are many limitations in the application of these drugs. Therefore, it is urgent to develop new treatments that can prevent or slow AD progression, for example, the inhibitors of KMTs and KDMs. In addition to medicine, environmental factors such as diet, physical activities, and stress, are all closely related to the development of AD. This review mainly focuses on the correlation between histone methylation, energy metabolism, and AD, so the search for dietary and lifestyle interventions that affect histone methylation or energy metabolism can be beneficial in advancing the translation of research findings into the clinical arena. For instance, curcumin was demonstrated to upregulate JMJD3 and reduce the level of H3K27me3 in the BDNF promoter region, improving mitochondrial function and reducing the level of Aβ [53]. Ascorbic acid serves as a potential coenzyme for specific histone demethylases of the JMJD family [176]. It was reported to have therapeutic benefits in addressing age-related diseases, such as AD [177, 178]. Sadli et al. [179] found that docosahexaenoic acid decreased concentrations of di-methylation of H3K4, H3K9, H3K27, H3K36, and H3K79 in M17 human neuronal cells. However, further research is necessary to determine whether ascorbic acid and docosahexaenoic acid have therapeutic effects on AD through histone methylation. Additionally, a recent study has discovered that oral supplementation with a multi-strain probiotic formulation reduced Aβ aggregates and brain damage by affecting lipid metabolism in a triple transgenic AD mouse model [180]. Reducing folate and vitamin B12 in neuroblastoma cell lines might also lead to a decrease in SAM levels, thereby increasing PS1 and BACE levels and Aβ production [181]. Apart from folate and vitamin B12, some micronutrients such as choline, methionine, and betaine, are essential for the generation of SAM, and they are proven to have important roles in ameliorating AD-like pathologies [182-184]. Therefore, increasing the amount of the above nutrients in the diet can be effective in preventing AD to some extent. Histone methylation can be dynamically regulated by stressors, including physical and psychological stress. A study using SAMP8 animals showed that chronic mild stress treatment increased H3K9 methylation [185]. Chronic social defeat stress in adult mice was found to significantly increase the levels of the H3K27me3 at the promoter of BDNF which is associated with synaptic plasticity and learning [186]. In terms of physical activities, exercise is associated with a lower risk for AD mortality. A previous study using APP/PS1 mice demonstrated that regular exercise alleviated cognitive impairment via increasing brain regional GLUT1- and GLUT3-mediated glucose metabolism [187]. Running exercise was reported to inhibit TREM2 shedding and maintain TREM2 protein levels, which were accompanied by the promotion of brain glucose metabolism, microglial glucose metabolism, and morphological plasticity in the hippocampus of AD mice [188]. Besides, Ying et al. [189] observed that amino acid metabolism was affected in the cortex and hypothalamus after high-intensity interval training in APP/PS1 mice. However, the relationship between lifestyle changes and histone methylation has not yet been clearly reported and needs to be further explored. Therefore, keeping a good diet, having regular exercises, and reducing stress can help to prevent and treat patients with AD.

## 6. Conclusion, limitations, and future directions

**Figure 3.**
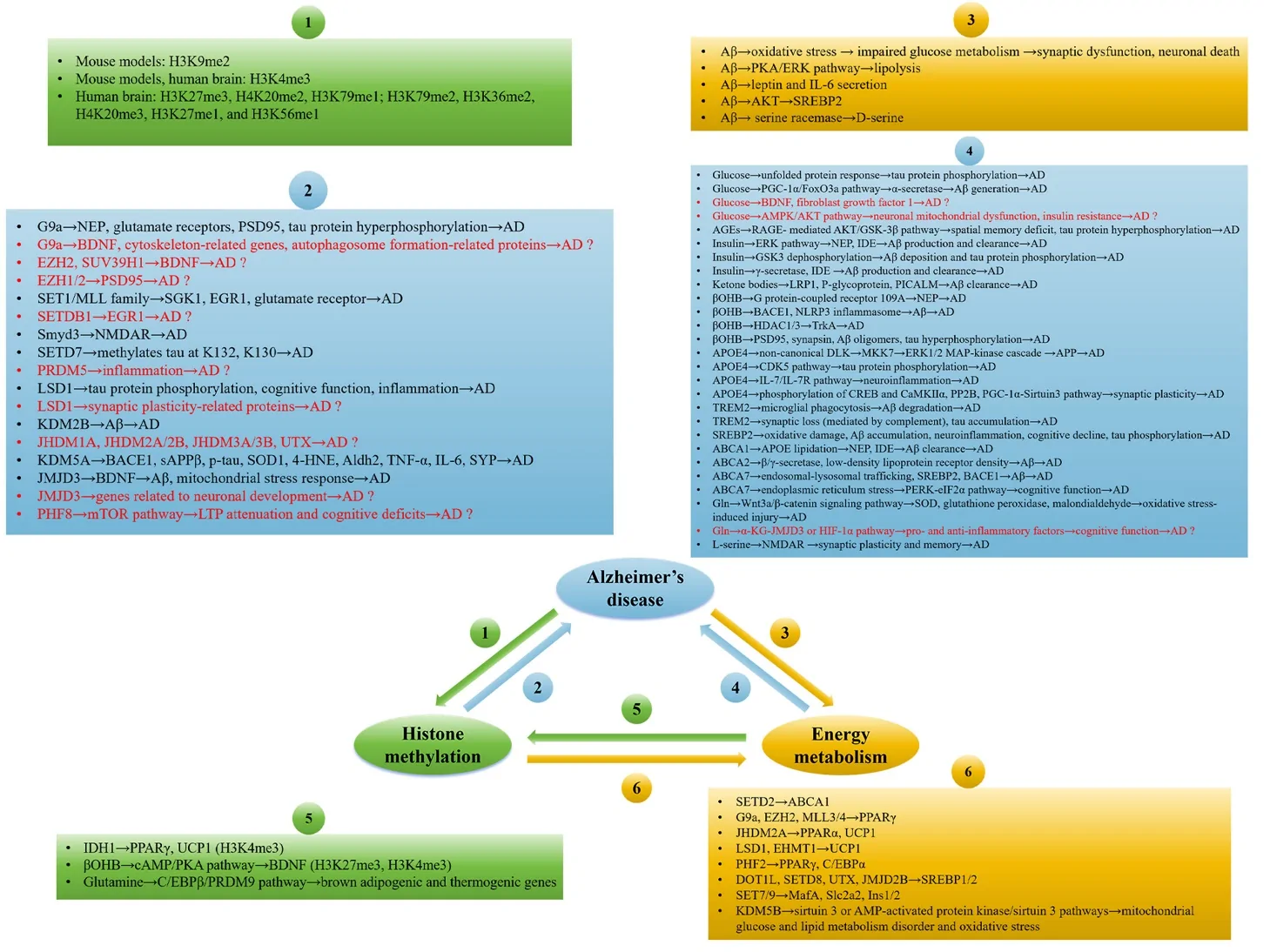
**The relationship and regulatory mechanisms of histone methylation, energy metabolism, and AD**. Note: The content not reported in AD is marked in red.

In this review, we have integrated recent studies on histone methylation, energy metabolism, and AD to better understand the relationship between the three of them (Fig. 3). Histone methylation, energy metabolism, and AD are closely associated with each other. Abnormal energy metabolism, especially in mitochondria, affects histone methylation by regulating the activities of relevant enzymes and the metabolism of intermediates. These enzymes and their intermediates promote or inhibit the expression of related genes, resulting in AD phenotypes. Various factors contributing to the pathological changes in AD, such as hypoxia, high ROS levels, calcium overload, and the pathological characteristics of AD itself, have been found to affect energy metabolism. Meanwhile, histone methylation regulates energy metabolism in the body. The interplay between histone methylation, energy metabolism, and AD is intricate and bidirectional. Further studies on their interactions may provide new insights into elucidating the pathogenesis of AD and controlling its progression.

However, there are some limitations remaining in this review. First, this review mainly focuses on the relationship between histone methylation and AD, but does not consider the effects of other types of histone modifications and the interactions of different types of histone modifications on pathological changes in AD. Second, in terms of AD treatment, this review concentrates on medication, diet, and lifestyle interventions targeting histone methylation and energy metabolism, however, the potential therapeutic targets of other epigenetic modification types have not been taken into consideration. Moreover, the studies included in the review are mainly based on *in vivo* and *in vitro* experiments with a low quality of evidence, so the specific roles of related indicators of histone methylation or energy metabolism in the brains of AD patients remain further explored.

Therefore, based on this review, there is still a lot of work to be done in the search for biomarkers, the innovation of mechanisms as well as experimental techniques and interventions, and the translation of research results in AD. Firstly, more in-depth studies on the effects of interactions between different epigenetic modifications, such as DNA methylation, histone modifications, and non-coding RNAs, on AD-related genes and signaling pathways. Secondly, the integration of multi-genomic approaches, such as genomics, transcriptomics, and proteomics with epigenetics, helps advance our understanding of AD. In recent years, with the advance of genetics, biochemistry, imaging and other disciplines, the combination of multiple disciplines can effectively improve the accuracy of early diagnosis of AD. For example, a research team used emerging technologies, such as artificial intelligence, to analyze CSF proteomics, and then identified novel biomarkers of important clinical value, which improves diagnostic accuracy and provides the possibility of early and precise diagnosis of AD [190]. Researchers have also found that the combination of participants’ electroencephalogram biomarkers, demographic information, CSF biomarkers, and APOE phenotype are effective in assessing individual cognitive function and disease progression [191]. In terms of treatment, advanced scientific methods, such as central targeting technology, nano drug delivery system, and deep brain stimulation, are used to overcome the difficulty of drugs penetrating and reaching the brain tissue and to control drug-resistant symptoms, so as to improve the therapeutic effect of drugs. Combining different types of therapies may provide a synergistic effect. For example, the integration of histone methylation or energy metabolism with conventional therapies, such as anti-amyloid treatments, and the combined therapies between epigenetic modifications and energy metabolism. It has also been suggested that a tight interlink between nutritional counseling, supervised physical training, oral health, and cognitive-oriented communication training would be beneficial in maintaining quality of life in AD patients [192]. Besides, attention has been given to the potential therapeutic targeting of other types of epigenetic modifications. More importantly, because the human organism exhibits higher complexity in answering to external factors, it is not easy to translate the findings from animal models into human interventions. Therefore, some of the findings mentioned in this review need to be confirmed by further clinical trials to reduce bias and enhance the reliability and credibility of the studies.

In conclusion, AD is the most common type of dementia. The pathogenesis of AD is influenced by multiple risk factors and is the result of the combined effects of multiple genes, multiple epigenetic modifications mechanisms, multiple energy metabolism pathways, and different brain regions. According to the latest report, effective invention on 14 risk factors, such as obesity, diabetes, air pollution, and lack of physical activities, may prevent or delay the occurrence of dementia by 45% [193]. Consequently, strengthening the communication and cooperation among various disciplines, exploring the mechanisms deeply by which risk factors affect AD, and adopting corresponding interventions to delay and prevent the occurrence of AD will be of great potential benefit to the aging population.
